# Fumarate induces redox-dependent senescence by modifying glutathione metabolism

**DOI:** 10.1038/ncomms7001

**Published:** 2015-01-23

**Authors:** Liang Zheng, Simone Cardaci, Livnat Jerby, Elaine D. MacKenzie, Marco Sciacovelli, T. Isaac Johnson, Edoardo Gaude, Ayala King, Joshua D. G. Leach, RuAngelie Edrada-Ebel, Ann Hedley, Nicholas A. Morrice, Gabriela Kalna, Karen Blyth, Eytan Ruppin, Christian Frezza, Eyal Gottlieb

**Affiliations:** 1 Cancer Research UK, Beatson Institute, Switchback Road, Glasgow G61 1BD, UK; 2 The Blavatnik School of Computer Science—Tel Aviv University, Tel Aviv 69978, Israel; 3 MRC Cancer Unit, University of Cambridge, Hutchison/MRC Research Centre, Box 197, Cambridge Biomedical Campus, Cambridge CB2 0XZ, UK; 4 Institute of Cancer Sciences, University of Glasgow, Switchback Road, Glasgow G61 1BD, UK; 5 School of Veterinary Medicine, College of Medical Veterinary and Life Sciences, University of Glasgow, Bearsden Road, Glasgow G61 1QH, UK; 6 Strathclyde Institute of Pharmacy and Biomedical Sciences, University of Strathclyde, 16 Richmond Street, Glasgow G1 1XQ, UK; 7 The Sackler School of Medicine—Tel Aviv University, Tel Aviv 69978, Israel

## Abstract

Mutations in the tricarboxylic acid (TCA) cycle enzyme fumarate hydratase (FH) are associated with a highly malignant form of renal cancer. We combined analytical chemistry and metabolic computational modelling to investigate the metabolic implications of FH loss in immortalized and primary mouse kidney cells. Here, we show that the accumulation of fumarate caused by the inactivation of FH leads to oxidative stress that is mediated by the formation of succinicGSH, a covalent adduct between fumarate and glutathione. Chronic succination of GSH, caused by the loss of FH, or by exogenous fumarate, leads to persistent oxidative stress and cellular senescence *in vitro* and *in vivo*. Importantly, the ablation of p21, a key mediator of senescence, in Fh1-deficient mice resulted in the transformation of benign renal cysts into a hyperplastic lesion, suggesting that fumarate-induced senescence needs to be bypassed for the initiation of renal cancers.

## Abstract (web summary)

Fumarate hydratase (FH) mutations are associated with renal cancer. Here, Zheng *et al*. use metabolomic and analytical chemistry approaches to reveal that fumarate accumulated due to FH loss covalently modifies intracellular glutathione, leading to oxidative stress and senescence.

## Graphical abstract

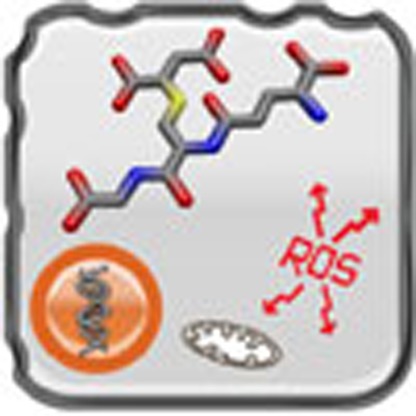

Fumarate hydratase (FH) mediates the reversible conversion of fumarate to malate in the tricarboxylic acid (TCA) cycle. The biallelic inactivation of FH leads to hereditary leiomyomatosis and renal cell cancer (HLRCC), a hereditary cancer syndrome characterized by the presence of benign tumours of the skin and uterus, and a highly malignant form of renal cell cancer^1^. Fumarate, which is highly accumulated in FH-deficient cells, has long been considered a major pro-oncogenic factor for HLRCC tumorigenesis^2^. It was initially proposed that the stabilization of the hypoxia-inducible factors (HIFs), caused by the fumarate-dependent inhibition of prolyl hydroxylases, was instrumental for tumour formation^3^,^4^. However, the recent findings that the genetic deletion of HIFs does not prevent cyst formation in Fh1-deficient mice^5^ challenged their aetiological role in these tumours, indicating that other HIF-independent oncogenic pathways are involved.

Fumarate is a moderately reactive α,β-unsaturated electrophilic metabolite that can covalently bind to cysteine residues of proteins under physiological conditions. This process, called protein succination, is a feature of FH-deficient tumour tissues, where fumarate accumulates to millimolar levels^6^. Keap1, an E3 ubiquitin ligase and an inhibitor of the transcription factor nuclear erythroid-related factor 2 (NRF2), has recently been identified as a target of protein succination and consequent inactivation, which led to the induction of NRF2 and a potent antioxidant response^5^,^7^. Of note, among the most characterized targets of NRF2, haem oxygenase 1 (HMOX1) has been found to be synthetic lethal with FH^8^ suggesting that the activation of NRF2 and its downstream targets might have an important prosurvival role in Fh1-deficient cancer cells, beyond the control of redox homeostasis.

Although Keap1 succination could explain the antioxidant transcriptional signature in FH-deficient tissues, other direct oxidation mechanisms could contribute to such a response. In fact, the loss of FH has been previously associated with the increased production of reactive oxygen species (ROS)^9^, and the accumulation of fumarate in FH-deficient cancer cell lines leads to the formation of an adduct between fumarate and glutathione (GSH), which depletes intracellular NADPH and enhances oxidative stress^10^. Nevertheless, a comprehensive investigation of the mechanism by which FH loss triggers oxidative stress, and the biological consequences of such stress, have not been performed.

Systems biology has become an invaluable tool for investigating cellular metabolism as it allows integrating data from different platforms such as transcriptomics and metabolomics. This approach was recently used to identify the molecular determinants of the Warburg effect^11^ and to explore novel therapeutic strategies for cancer such as metabolic synthetic lethality^12^. In the present work, we used a combination of computational biology, metabolomics and mouse genetics to investigate the overall metabolic effects of FH loss and, in particular, the possible outcomes of FH loss and oxidative stress. We demonstrated that the accumulated fumarate leads to oxidative stress by covalently binding to reduced GSH in a non-enzymatic reaction that forms succinicGSH. We also demonstrated that the oxidative stress induced by GSH succination is necessary and sufficient to elicit cellular senescence in non-transformed cells and that the ablation of senescence can initiate a tumorigenic programme in the kidneys of Fh1-deficient mice.

## Results

### Oxidative stress in FH-deficient cells

**Figure 1:**
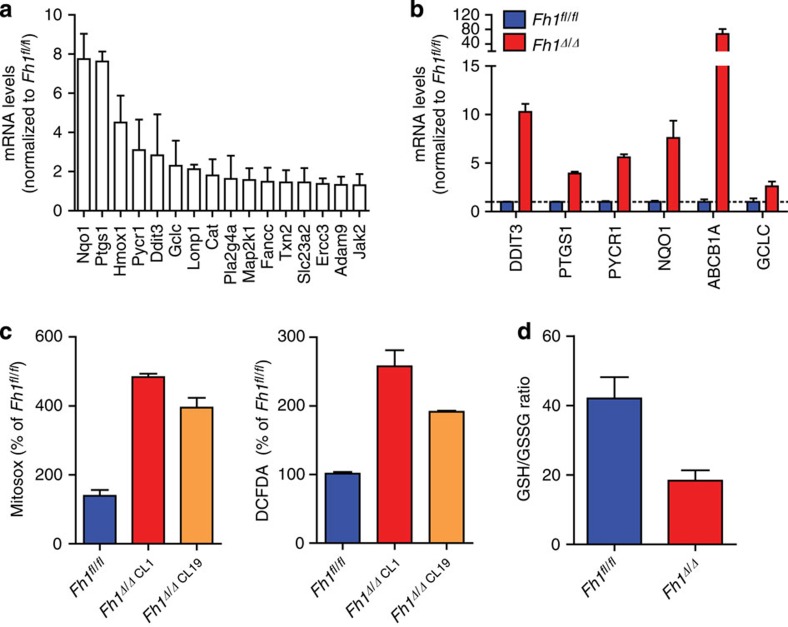
Antioxidant signature in FH-deficient cells. (**a**,**b**) Expression of genes that belong to ‘antioxidant response’ retrieved from a gene expression array (**a**) and validated by qPCR (**b**) from FH-deficient mouse kidney epithelial, as compared with control cells. qPCR data were obtained from three independent cultures and represented as average±s.e.m. (**c**,**d**) FH-deficient cells exhibit increased oxidative stress measured by mitosox (**c**), DCFDA (**c**) and by decreased GSH/GSSG ratio (**d**). Results were obtained from three independent experiments and represented as average±s.e.m.

To elucidate the metabolic changes that occur on the loss of FH, we utilized the previously characterized mouse FH-deficient kidney cells (*Fh1*^*Δ/Δ*^) and their wild-type (wt) isogenic control (*Fh1*^*fl/fl*^) (ref. 8). Initially, we found that genes whose expression is upregulated in FH-deficient cells^8^ are significantly enriched with oxidative stress genes (hypergeometric *P* value of 5.505e−04, see Methods). Reassuringly, genes that are upregulated in HLRCC clinical samples relative to normal kidney tissues^7^ are also significantly enriched with oxidative stress genes (hypergeometric *P* value of 3.984e−06). The genes that featured most highly (Fig. 1a) were then validated by quantitative PCR (qPCR; Fig. 1b). In line with this oxidative stress signature, Fh1-deficient cells exhibit increased ROS production and a substantial drop in GSH/GSSG ratio (Fig. 1c,d).

To better understand the metabolic consequences of FH loss, we performed a comprehensive analysis of extracellular metabolite exchange rates using liquid chromatography-mass spectrometry (LC-MS; Supplementary Table 1). We then generated *in silico* genome-scale metabolic models of the FH-deficient and wt cells by incorporating the gene expression and metabolite exchange rates measured in these cell lines within a generic metabolic model^13^ (see Methods). Of note, these implemented models not only explore essential reactions for biomass production, as previously done^8^, but also allowed us to predict changes in metabolic fluxes on loss of FH. We first examined the models by testing their ability to predict the measured flux rates via cross-validation (leaving one out). For each iteration, we regenerated a new model based on the gene expression and a partial set of the metabolite uptake and secretion rates and used these models to predict the flux rate through the reaction whose flux measurement was omitted. Significant correlation was found between the measured and predicted flux rates (*R*=0.617 and 0.551, *P* values of 6.59e−04, and 2.63e−03, when testing the wt and FH-deficient models, respectively Supplementary Table 2).

Following the validation of the models, we utilized them to systematically explore the metabolic differences between the FH-deficient and control cells. We computed the capacity of the two models to produce each of the 1,491 metabolites included in the models. The FH-deficient model was found to have a higher capacity to produce 43 metabolites. Among the top ones are four derivatives of GSH (Supplementary Fig. 1a,b). Furthermore, GSH biosynthesis was projected to be an essential metabolic reaction in the Fh1-deficient model (Supplementary Fig. 3b). We also predicted that the FH-deficient model has a significantly lower capacity to produce reducing power, in the form of NADH and NADPH, compared with the wt model (Supplementary Fig. 1c). Of note, in the computational model, the loss of HMOX1, whose essentiality in the Fh1-deficient cells has been previously shown^8^, was expected to exacerbate the decreased ability of the FH-deficient cells to produce NADPH.

### Metabolomics revealed GSH succination in FH-deficient cells

**Figure 2:**
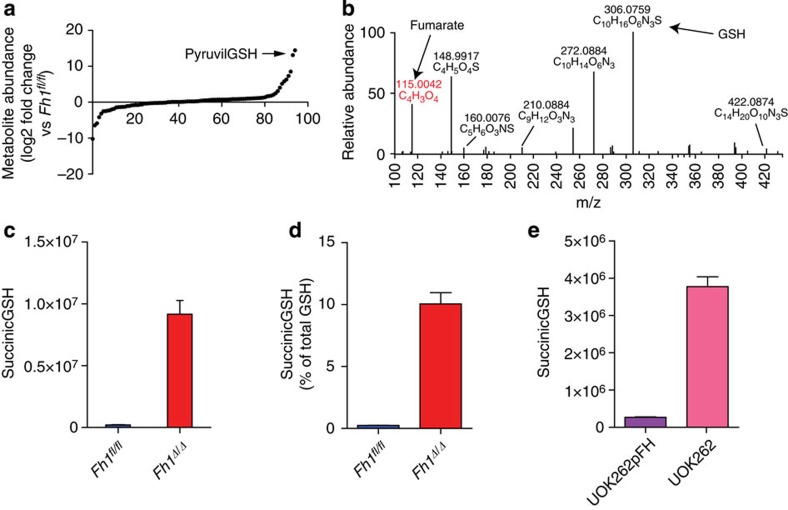
Accumulation of fumarate leads to GSH succination. (**a**) Relative abundance of intracellular metabolites in FH-deficient cells measured by LC-MS. Results were obtained from nine independent cultures. (**b**) Fragmentation pattern of the metabolite annotated as pyruvilGSH obtained by LC-MS/MS. (**c**–**e**) Intracellular abundance of succinicGSH in mouse (**c**) and human (**e**) FH-deficient cells. (**d**) Amount of succinicGSH compared with the pool of GSH in mouse cells. succinicGSH and GSH were measured by LC-MS. Results were obtained from three independent experiments and represented as average±s.e.m.

To further confirm that GSH metabolism was altered in FH-deficient cells an LC-MS analysis of steady-state intracellular metabolites was performed. Among the most significant metabolites accumulated in FH-deficient cells a putative GSH adduct was detected (annotated by Metlin database as the formate adduct of pyruvilGSH; Fig. 2a). Since this metabolite is specific to FH-deficient cells and is poorly characterized, we decided to elucidate its structure by LC-MS/MS and NMR analyses. The MS/MS fragmentation pattern and the presence of fumarate and GSH fragments as diagnostic daughter ions (Fig. 2b), strongly indicated that this metabolite was wrongly annotated and is instead an adduct of fumarate binding to GSH, which we defined as succinicGSH (Supplementary Fig. 2a for the chemical structure). Furthermore, NMR pulse-field gradient correlation spectroscopy (pfgCOSY), pulse-field gradient total correlation spectroscopy (pfgTOCSY; Supplementary Fig. 2b) and further LC-MS/MS analysis (Supplementary Fig. 2c) confirmed the chemical structure of this molecule (registered as S-(1,2-Dicarboxyethyl)glutathione: CAS Registry Number [1115-52-2]).

After elucidating the molecular structure of succinicGSH, we confirmed its presence in *Fh1*^*Δ/Δ*^ cells using targeted LC-MS, showing that succinicGSH represents about 10% of the total GSH in these cells (Fig. 2c,d). It is noteworthy that succinicGSH was also recently characterized in the human FH-deficient renal cancer cell line UOK262 (ref. 10) and we confirmed its presence in these cells (Fig. 2e).

### SuccinicGSH is formed by a reaction of fumarate with GSH

**Figure 3:**
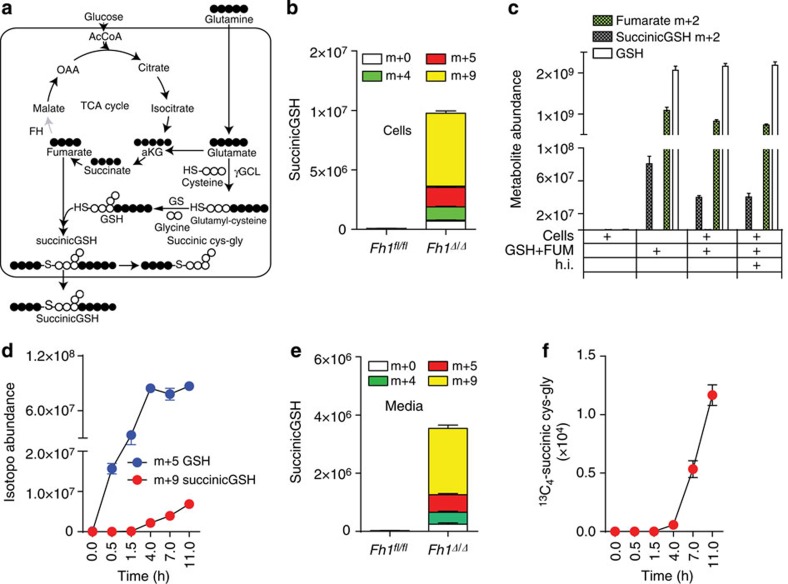
The formation and fate of succinicGSH. (**a**) Schematic representation of GSH biosynthesis in FH-deficient cells on incubation with ^13^C_5_-glutamine. The white and black circles under each metabolite illustrate ^12^C- and ^13^C-, respectively. (**b**) Isotopologue distribution of succinicGSH was measured by LC-MS after incubating cells of the indicated genotype in the presence of ^13^C_5_-glutamine for 8 h. (**c**) GSH and ^13^C_2_-fumarate were mixed in a physiological buffer in the presence or absence of protein lysates from FH-deficient cells and the amount of ^13^C_2_-succinicGSH was measured by LC-MS. Where indicated, the cell extracts were heat inactivated (h.i.) before the *in vitro* reaction. (**d**) The kinetics of GSH and succinicGSH formation from ^13^C_5_-Glutamine. (**e**) The relative level of succinicGSH in the media of mouse cells with the indicated genotype. (**f**) The kinetics of the formation of the degradation product from GSH was traced by ^13^C_5_-Glutamine and assessed by LC-MS. Results were obtained from three independent experiments and represented as average±s.e.m.

To further elaborate on the source of succinicGSH in FH-deficient cells, we tested the hypothesis that this compound is formed by a reaction between accumulated fumarate and GSH. To this end, FH-deficient cells were incubated with uniformly labelled ^13^C_5_-glutamine, which generates ^13^C_5_-labelled glutamate and ^13^C_4_-labelled fumarate, and the isotopologue distribution of succinicGSH was analysed by LC-MS (see Fig. 3a for a schematic diagram of the reactions). SuccinicGSH isotopologues were consistent with carbons coming mostly from fumarate (*m*+4) and glutamate (*m*+5), which eventually gave rise to a *m*+9-labelled metabolite (Fig. 3b).

At physiological pH, the thiol residue of GSH is scarcely deprotonated (pK_a_=9.2). Therefore, the reactions between GSH and electrophilic molecules are generally catalysed by enzymes, such as GSH transferases, which activate GSH by ionizing its thiol group (SH) into the more reactive thiolate (S^−^) residue^14^. We therefore wanted to address whether the production of succinicGSH required an enzymatic reaction. To this end, GSH and fumarate, at a concentration found in Fh1-deficient cells^15^, were mixed in a physiological buffer. To avoid interference from endogenous fumarate and succinicGSH from cell extracts, ^13^C_2_-fumarate was used. When labelled fumarate and GSH were mixed in a physiological buffer, a significant amount of m+2 labelled succinicGSH was detected (Fig. 3c). The addition of cell protein extracts from Fh1-deficient cells did not increase and, heat inactivation did not decrease the production of succinicGSH, indicating that the formation of the compound under these conditions is non-enzymatic. Together these results demonstrated that fumarate, when accumulated, leads to GSH succination.

We further investigated the rate of succinicGSH formation and its degradation. To this aim, Fh1-deficient cells were incubated with ^13^C_5_-glutamine and the kinetics of succinicGSH formation was monitored. The labelling of succinicGSH occurred very rapidly on incubation with labelled glutamine and it tailed GSH biosynthesis (Fig. 3d). Of note, we could not detect the succinated form of the GSH precursor *γ*-glutamylcysteine in Fh1-deficient cells, suggesting that GSH, rather than one of its precursors, is subject to succination. We finally investigated the fate of succinicGSH. Experiments on Fh1-deficient cells incubated with ^13^C_5_-glutamine revealed that succinicGSH is excreted in the media (Fig. 3e) and its isotopologue labelling matches that of intracellular succinicGSH (Fig. 3b). Importantly, in these cells, we have also identified a succinated form of the dipeptide cys–gly, the degradation product of GSH. The rate of production of this metabolite, which followed both GSH and succinicGSH kinetics (Fig. 3f), suggests that succinic cys–gly is likely a breakdown product of succinicGSH. Together this investigation revealed that succinicGSH is formed by succination of newly synthesized GSH and that this metabolite is partly degraded into succinated cys–gly and partly secreted to the media (See Fig. 3a for a schematic of these reactions).

### Succination of GSH increases oxidative stress

**Figure 4:**
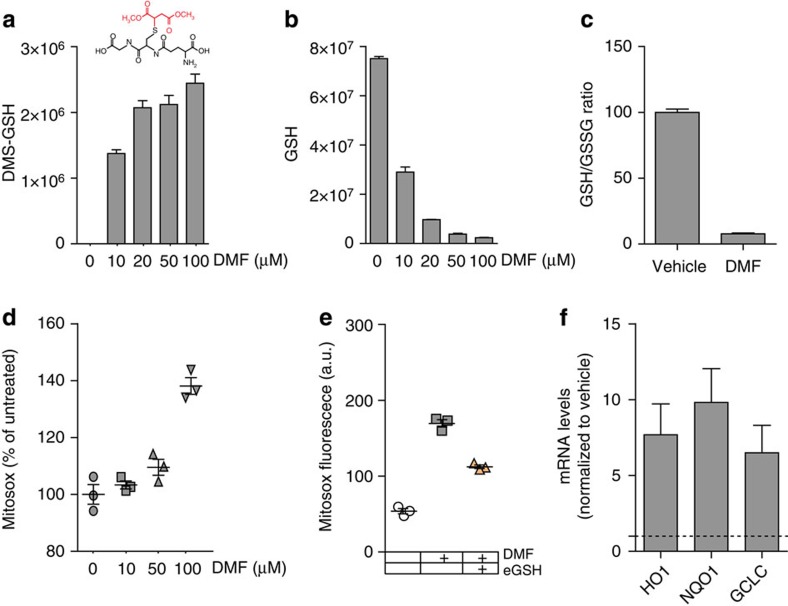
DMF induces GSH succination and increases oxidative stress. (**a**–**c**) Cell-permeable DMF binds to (**a**) and depletes (**b**) the intracellular pool of GSH, leading to a decrease in GSH/GSSG ratio (**c**). The structure of the adduct between GSH and DMF, dimethyl-succinicGSH (DMS-GSH), is indicated in **a**. (**d**–**f**) DMF induces oxidative stress (**d**,**e**) and the activation of an antioxidant response, measured by the expression of antioxidant genes (**f**). The redox stress caused by DMF is attenuated by incubating cells with a cell-permeable derivative of GSH, ethyl-GSH (**e**). GCLC, glutamate-cysteine ligase; HO1, haem oxygenase1; NQO1, NAD(P)H dehydrogenase (quinone 1). Results were obtained from three independent experiments and represented as average±s.e.m.

We wanted to investigate the link between GSH succination and the oxidative stress signature that characterize FH-deficient cells (Fig. 1). Sullivan *et al*.^10^ previously proposed that enhanced oxidative stress in these cells is caused by the NADPH-depleting conversion of succinicGSH into GSH, a reaction catalysed by GSH reductase (GR). However, this conclusion was based on indirect evidence, that is, consumption of NADPH on incubation of a methylated version of succinicGSH at high concentration and GR^10^. To test their hypothesis, we measured the activity of GR towards chemically synthesized succinicGSH as compared to its natural substrate oxidized GSH (GSSG) by measuring the products. While GSSG was quickly reduced to GSH, we could not detect GSH production from succinicGSH in the presence of GR (Supplementary Fig. 3a). Therefore, it is unlikely that the detoxification of succinicGSH by GR provides explanation for the enhanced redox stress in Fh1-deficient cells. Several other factors could contribute to the oxidative stress in these cells, including electron transport chain dysfunction, as previously documented^16^. Therefore, to investigate the mechanistic link between fumarate accumulation, succination of GSH, and oxidative stress, respiration-competent *Fh1*^*fl/fl*^ cells were incubated with a cell-permeable derivative of fumarate, dimethylfumarate (DMF). DMF quickly reacted with intracellular GSH leading to the formation of dimethyl-succinicGSH (Fig. 4a). Of note, the incubation with DMF caused a dose-dependent depletion of GSH (Fig. 4b), a substantial drop in the GSH/GSSG ratio (Fig. 4c) and increased oxidative stress (Fig. 4d,e). Furthermore, DMF treatment elicited an antioxidant gene response similar to that observed in *Fh1*^*Δ/Δ*^ cells (Fig. 4f). Importantly, the incubation with a cell-permeable derivative of GSH, ethyl-GSH, significantly decreased the ROS production triggered by DMF (Fig. 4e). Together these results indicate that the succination of GSH, but not its detoxification, is responsible for the oxidative stress observed in Fh1-deficient cells

### GSH succination increases GSH biosynthesis

**Figure 5:**
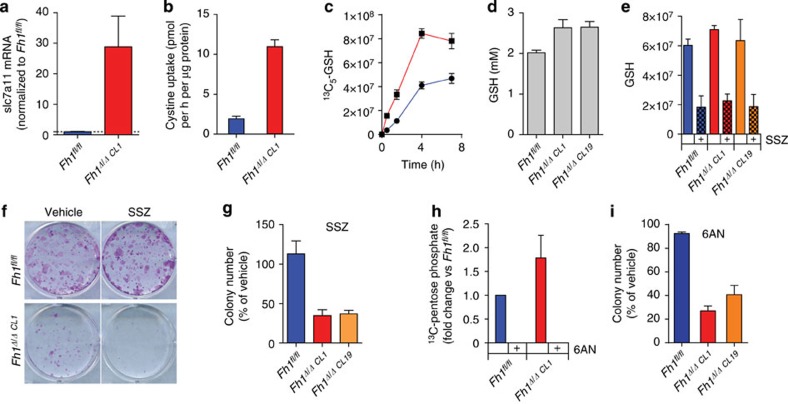
GSH succination triggers cystine uptake and GSH biosynthesis. (**a**–**c**) The loss of FH leads to the upregulation of the cystine transporter (**a**), increased cystine uptake (**b**) and accelerated GSH biosynthesis traced from ^13^C_5_-glutamine (**c**). (**d**) The steady-state pool of GSH is increased in FH-deficient cells. (**e**,**f**,**g**) Sulfasalazin (SSZ) inhibits GSH biosynthesis in both wt and FH-deficient cells (**e**) but selectively impairs cell survival and growth of FH-deficient cells (**f**,**g**). (**h**,**i**) 6-Aminonicotinamide (6-AN), an inhibitor of the PPP, inhibits pentose phosphate production in both cell lines as assessed by tracing ^13^C_6_-Glucose (**h**), selectively affects the proliferation of FH-deficient cells (**i**). The images in **f** are the representative images of triplicate cultures. Results were obtained from three independent experiments and represented as average±s.e.m.

We hypothesized that the chronic depletion of GSH caused by succination could perturb cell metabolism and contribute to a general deregulation of redox homeostasis. To address this question, we reconstructed the metabolic models previously generated to include GSH succination. Of note, the inclusion of this reaction improved the ability of the FH-deficient model to predict the measured flux rates (*R*=0.570, *P*=1.81E−03; Supplementary Table 2). We found that in the FH-deficient model, but not in the wt model, GSH succination significantly reshaped the generation of reducing power (Supplementary Fig. 1b). Furthermore, the model revealed that GSH succination impinged GSH metabolism and cystine uptake (Supplementary Table 3). Notably, the selective uptake of cystine (an oxidized dimer of cysteine), rather than methionine, as a precursor for cysteine and subsequently GSH biosynthesis imposes a further metabolic constraint: the reduction of cystine to cysteine requires NADPH, the supply of which becomes critical for GSH biosynthesis (see Supplementary Fig. 3b). In line with these predictions, *Fh1*^*Δ/Δ*^ cells exhibited a significant overexpression of Slc7a11, a member of the cystine transporter xCT, which was functionally associated with increased uptake of cystine (Fig. 5a,b). The rate-limiting step in GSH biosynthesis is the synthesis of γ-glutamylcysteine from glutamate and cysteine by γ-glutamylcysteine synthetase (GCL; see Fig. 3a for a schematic representation of these reactions). GSH is then synthesized by GSH synthetase (GSS), which adds glycine to γ-glutamylcysteine. Therefore, the GSH biosynthesis rate could be assessed by the rate of incorporation of glutamine-derived glutamate after incubation with ^13^C_5_-glutamine. The incorporation of glutamate into GSH was faster in FH-deficient cells, meaning that, as predicted by the computational models, GSH production is accelerated in FH-deficient cells (Fig. 5c). Moreover, the increased biosynthesis rate of GSH caused by GSH succination led to a larger steady-state pool of GSH (Fig. 5d). In line with these findings, genes encoding for cystine transport (Slc7a11) and enzymes of the GSH biosynthetic pathway (GSS and GCL), are significantly upregulated in HLRCC patients (Supplementary Fig. 4)^7^,^17^. All in all, these suggest that increased GSH biosynthesis could be a compensatory mechanism that mitigates the increased oxidative stress in FH-deficient cells.

To test whether GSH biosynthesis is required for the survival of FH-deficient cells, we inhibited cystine uptake using a well-characterized xCT inhibitor, sulfasalazine. Reassuringly, the inhibition of cystine uptake significantly impaired GSH biosynthesis in both genotypes (Fig. 5e). However, only *Fh1*^*Δ/Δ*^ cells were negatively affected by the drug (Fig. 5f). Since GSH biosynthesis requires NADPH supply to reduce cystine to cysteine (Supplementary Fig. 3b), we tested the effects of inhibiting NAPDH production on the survival of FH-deficient cells. The pentose phosphate pathway (PPP) is the major supply of NADPH in cells and so cells were incubated with 6-amino nicotinamide, a known inhibitor of the PPP enzyme 6-phosphogluconate dehydrogenase. 6-Amino nicotinamide effectively halted PPP activity in both genotypes (Fig. 5g) but it only affected the survival of FH-deficient cells (Fig. 5h). In summary, these results indicate that GSH biosynthesis is essential for FH-deficient cells to overcome the oxidative stress caused by GSH succination.

### Loss of FH leads to oxidative stress-mediated senescence

**Figure 6:**
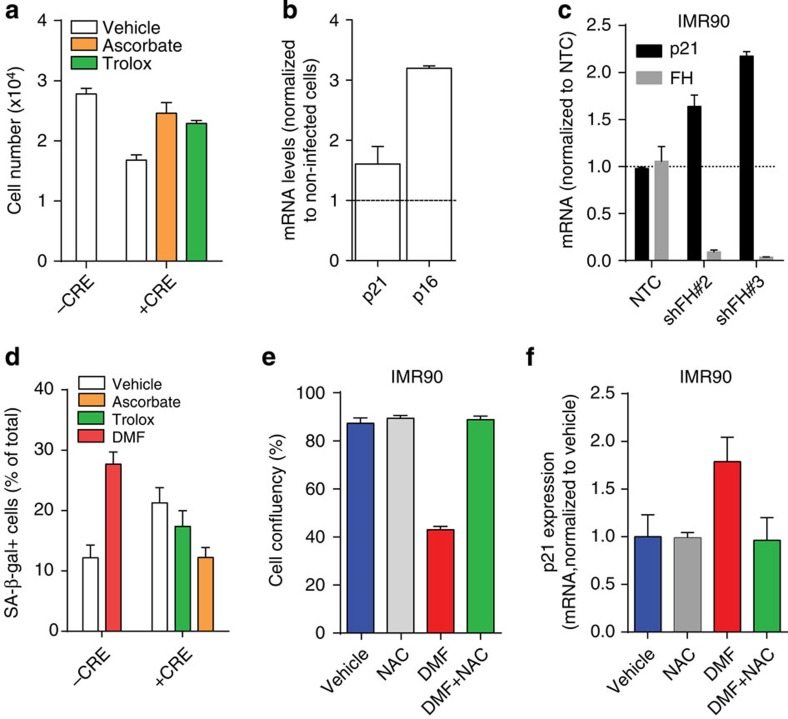
GSH succination leads to redox stress-induced senescence in primary kidney cells and human diploid fibroblasts. (**a**) Primary epithelial kidney cells (1 × 10^4^) from *Fh1*^*fl/fl*^ animals were either left uninfected or infected with adeno-CRE and treated with vehicle or the antioxidant trolox or ascorbate. Cell number was measured after 2 days of culture. (**b**) The ablation of FH in cells described in **a** resulted in the activation of p16 and p21 transcription. Results are expressed as average fold induction over non-infected cells±s.e.m (representative experiments; *n*=6 for p21 and *n*=3 for p16). (**c**) FH and p21 mRNA levels were assessed by qPCR in non-targeting control (NTC)-transfected cells in FH-silenced cells. (**d**) Microscopy quantitative analysis of senescence-associated β-galactosidase activity, induced by DMF or adeno-CRE infection, with or without trolox and ascorbate. (**e**,**f**) cell growth (**e**) and p21 mRNA expression levels (**f**) were assessed in IMR90 cells after DMF treatment, with or without the antioxidant *N*-acetyl cysteine (NAC). All the results were obtained from three independent cultures and expressed as average±s.e.m.

We hypothesized that the observed metabolic reprogramming is a required step for the support of cell transformation. Since both the human and mouse FH-deficient cells used here were immortalized cell lines, we tested the effects of GSH succination on primary kidney cells isolated from *Fh1*^*fl/fl*^ mouse pups and on the human diploid fibroblasts IMR90. On infection with adenoviral vector expressing Cre recombinase (Ad-Cre), *Fh1*^*fl/fl*^ cells, which lost FH activity, underwent proliferation arrest, whereas the uninfected cells continued to proliferate (Fig. 6a). The proliferation arrest was associated with a robust induction of the cyclin-dependent kinase inhibitor p21^CIP1/WAF1^ (thereafter referred to as p21) and of the cyclin-dependent kinase inhibitor 2A p16 (Fig. 6b). These results suggested that oxidative stress-mediated cellular senescence^18^ might be responsible for the observed phenotype. Similarly, the non-transformed non-immortalized human diploid fibroblast cells IMR90 exhibited a significant upregulation of p21 on acute depletion of FH (Fig. 6c). To confirm the involvement of senescence, we tested the senescence-associated β-galactosidase (SA-β-gal) activity in FH-deficient cells. On infection with Ad-Cre, *Fh1*^*fl/fl*^ exhibited a strong SA-β-gal staining (Fig. 6d and Supplementary Fig. 5a). Reassuringly, the loss of FH was also associated with the activation of an antioxidant response similar to that observed in Fh1-deficient cell lines (Supplementary Fig 5b). To ascertain the role of oxidative stress in senescence induction, cells were incubated with ascorbate (vitamin C) or Trolox (vitamin E derivative), two well-characterized antioxidants, which are water or lipid soluble respectively. Importantly, both the growth inhibitory effect, and the induction of SA-β-gal activity in Fh1-deficient primary kidney cells were abrogated by these antioxidants (Fig. 6a,d). These results indicate that redox stress induced by FH loss is, at least in part, responsible for the ensuing senescence.

We then wanted to test the direct role of GSH succination in fumarate-induced senescence. Primary kidney cells and IMR90 cells were treated with DMF, a potent inducer of succination-dependent oxidative stress and their proliferation was assessed. DMF elicited a dose-dependent inhibition of cell proliferation and induced SA-β-gal activity in non-transformed primary mouse kidney epithelial cells (Fig. 6d and Supplementary Fig. 5a,c). Consistent with these results, DMF-treated IMR90 exhibited profound proliferation defects (Fig. 6e and Supplementary Fig. 5d), p21 upregulation (Fig. 6f) and SA-β-gal positivity (Supplementary Fig. 5d), which were rescued by the incubation with the antioxidant *N*-acetyl cysteine (Fig. 6e,f and Supplementary Fig. 5d,e). These results strongly suggest that fumarate-induced oxidative stress is sufficient for the induction of p21 and the initiation of cell senescence, regardless of cells’ genotype.

### Fumarate-induced senescence in renal cysts of *Fh1* ^*Δ/Δ*^ mice

**Figure 7:**
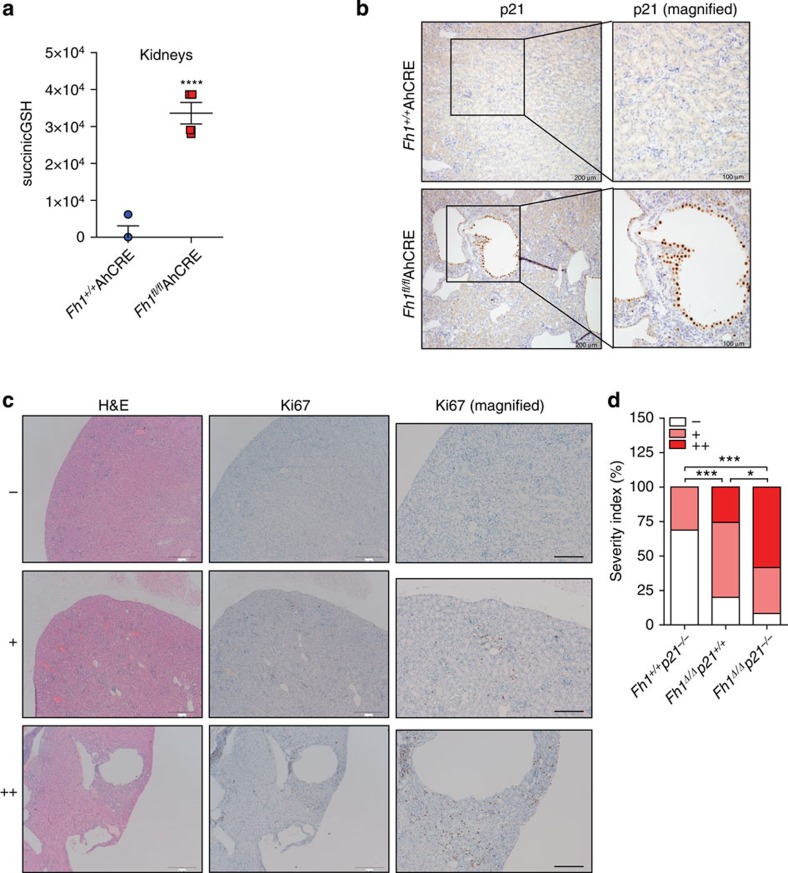
GSH succination is associated with fumarate-induced senescence in renal cysts. (**a**) SuccinicGSH was measured in kidney extracts of the indicated animals by LC-MS. Four animals for each genotype were used for this experiment. (**b**) Representative images of p21 immunostaining performed on dissected kidneys of the indicated mice. (**c**) Representative haemotoxylin and eosin staining and Ki67 immunostaining images of the different histological grading of kidney hyperplasia observed in 12–18-month-old mice. Representative images from normal kidneys, scored as (−), were taken from Fh1^+/+^ p21^−/−^ animals; kidneys with limited number of cysts (arrows) and areas of hyperplasia with low overall Ki67, staining scored as (+), were taken from Fh1^*Δ*/*Δ*^ p21^+/+^ animals; polycystic kidneys with increased Ki67 immunostaining mainly localized around large cystic areas (arrows), scored as (++), were taken from Fh1^*Δ*/*Δ*^ p21^−/−^. Scale bars represent 500 μm and 200 μm respectively. (**d**) Quantitative analysis of the % distribution of the different hyperplastic grades described in **c** in the indicated genotypes. The results are derived from *n*=32 of Fh1^+/+^ p21^−/−^, *n*=35 of Fh1^*Δ/Δ*^ p21^+/+^ and *n*=24 of Fh1^*Δ/Δ*^ p21^−/−^ mice aged 12–18 months. *P* values calculated by *χ*^2^-analysis are indicated (***P<0.0001, *P<0.05).

Previous work demonstrated that the loss of FH in murine kidneys does not result in overt renal carcinomas, as observed in HLRCC patients, but rather in benign cysts^19^. Our results indicated that the redox stress mediated by the accumulation of fumarate caused by the loss of Fh1 leads to senescence. Therefore, we hypothesized that senescence could be a major adjournment event of the tumorigenic process in *Fh1*-deficient animals. To test this hypothesis, we used the previously characterized *Fh1*^*fl/fl*^*AhCre* mice, which develop FH-deficient renal cysts^15^. Metabolite extraction confirmed the presence of high levels of succinicGSH in the kidneys of these animals (Fig. 7a). In line with our findings in FH-deficient cell lines, many of the epithelial cells facing the lumen of the renal cysts showed an intense staining for p21 (Fig. 7b). To address the role of p21 in the suppression of tumorigenesis in Fh1-null mice, we crossed *Fh1*^*fl/fl*^*AhCre* with p21^−/−^ mice, obtaining animals deficient for both Fh1 and p21 in the kidneys (*Fh1*^*Δ/Δ*^*p21*^*−/−*^). Histological analyses revealed that a simultaneous ablation of these genes resulted in the presence of several hyperproliferative areas in the kidneys. This was assessed by haematoxylin and eosin staining for cellular density and by Ki67 for proliferative capacity of the cells^20^. Strikingly, several areas of the kidneys of *Fh1*^*Δ/Δ*^*p21*^*−/−*^ animals were characterized by strong Ki67 staining (Fig. 7c ++; quantified in Fig. 7d). In fact, 14 out of 24 (58.33%) of the *Fh1*^*Δ/Δ*^*p21*^*−/−*^animals exhibited dense areas with proliferative and locally invasive kidney epithelial cells (cytokeratin positive) (Fig. 7c ++ and Supplementary Fig. 6). These results strongly suggest that the bypass of senescence may initiate a tumorigenic programme in *Fh1*-deficient mice.

## Discussion

FH is emerging as an important mitochondrial tumour suppressor in both hereditary and sporadic forms of cancer^2^. Although FH mutations in HLRCC were discovered more than a decade ago, the specific consequences of the loss of this TCA cycle enzyme remained unclear. The biochemical consequences of Fh1 inactivation in both human and mouse cells have been extensively investigated, revealing a complex pattern of metabolic defects, including deregulation of TCA and urea cycle^15^. Nevertheless, a global analysis of the metabolic rewiring on the loss of Fh1 is still missing. To address this question, we generated *in silico* models to explore the metabolic consequences of FH inactivation. These models, which integrate transcriptomics and consumption-release metabolomics data to predict metabolic fluxes, revealed unexpected metabolic features of FH-deficient cells. Interestingly, we found that a large number of metabolic pathways became essential on the loss of FH, among which haem biosynthesis and degradation pathway and GSH biosynthesis were the most enriched. While the haem biosynthesis and degradation pathway has already been demonstrated to be synthetic lethal in FH-deficient cells, the role of GSH metabolism in FH-deficient cells has not been investigated before and required further investigation. An untargeted intracellular metabolomics analysis helped to shed light on these findings. We found that the aberrant accumulation of fumarate leads to the persistent depletion of GSH via the formation of the adduct succinicGSH. We confirmed that succinicGSH, which is all but absent from normal cells, accumulates in both human and mouse FH-deficient cells and in the kidneys of *Fh1*-deficient animals, suggesting that this metabolite could be used as a beacon for toxic accumulation of fumarate. In the work presented here, we showed that succinicGSH is formed by a non-enzymatic reaction between fumarate and the thiol residue of *de novo* biosynthesised GSH. Furthermore, we found that succinicGSH is secreted by FH-deficient cells and can be degraded, in a process similar to GSH catabolism, into succinic cys–gly.

SuccinicGSH has been recently identified in the human FH-deficient cancer cell line UOK262 (ref. 10). In this work, Sullivan *et al*.^10^ proposed that the detoxification of succinicGSH mediated by GR explains the enhanced oxidative stress in FH-deficient cells . However, our data showed that the contribution of GR to succinicGSH degradation is minor and unlikely to account for increased oxidative stress in these cells. Instead, we found that succination of GSH significantly increases the demands of NADPH to sustain GSH biosynthesis from cysteine. This reaction negatively affects redox homeostasis and, as a compensatory mechanism, FH-deficient cells enhance cystine uptake and GSH biosynthesis. Therefore, although FH-deficient cells experience a chronic oxidative stress, GSH levels are not compromised in these cells. How these cells genetically orchestrate this metabolic reprogramming is not known. The accumulation of fumarate in FH-deficient human cancers has been previously linked with the activation of the transcription factor Nrf2 (refs 5, 7), which elicits an antioxidant response and prevents the elevation of ROS to toxic levels in these cells. Keap1 is also regulated by oxidation of its thiol residues^21^; therefore, the increased oxidative stress observed in FH-deficient cells could also contribute to Nrf2 activation. We hypothesize that both the redox stress caused by GSH succination and Keap1 succination contribute to the emergence of the antioxidant signature and Nrf2 activation to support GSH biosynthesis and to maintain a viable level of oxidative stress.

Persistent oxidative stress has been previously linked to the induction of senescence^18^. We reasoned that overt oxidative stress caused by the loss of FH in non-transformed cells could instigate a tumour suppressive mechanism that could explain why *Fh1*-deficient animals do not develop carcinomas but only benign cysts^19^. To accurately dissect the effects of accumulation of fumarate in early phases of epithelial transformation, we silenced FH in non-immortalized non-transformed cells. In primary epithelial kidney cells and in human diploid fibroblasts, the loss of FH or incubation with DMF leads to a robust induction of senescence, confirmed by proliferative arrest, p21 upregulation and SA-β-galactosidase activity. The incubation with antioxidants abrogated the anti-proliferative response, suggesting that the oxidative stress induced by fumarate accumulation is responsible for the induction of senescence. In line with these results, we observed that some renal cysts exhibit a strong positive staining for p21, indicating that senescence is also a factor that may prevent or delay malignant transformation *in vivo*. In line with this notion, the co-ablation of p21 and Fh1 resulted in high proportion of hyperprolifearative areas in the kidney. Importantly, p21, but not p16 induction is also observed in human HLRCC patients (Supplementary Fig. 4). These results confirm the association between FH loss and p21 induction in human tumours. Furthermore, they may indicate that in human FH-deficient renal cancer, the lack of p16 induction may help avoiding the senescence phenotype.

The observation that the loss of FH, a bona fide tumour suppressor gene^22^, could lead to senescence, although counterintuitive, suggests that the role of FH in tumorigenesis is complex and might involve secondary oncogenic alterations to overcome senescence. It is worth mentioning that the loss of FH is not the only example of an oncogenic event that leads to senescence at early stages of tumorigenesis. Other examples include the loss of the tumour suppressors VHL and PTEN^23^,^24^ as well as the activation of the oncogenes Ras or BRAF^25^,^26^,^27^. Although the underlying mechanisms that lead to senescence within these models might be different, a general accepted paradigm is that the unrestrained proliferation elicited by an oncogenic event leads to the activation of tumour restraining events. In this work we demonstrated that in the case of FH loss, this activity is mediated by fumarate, at least in part via GSH succination.

## Methods

### Animal work

Animal work was carried out with ethical approval from University of Glasgow under the Animal (Scientific Procedures) Act 1986 and the EU Directive 2010 (PPL 60/4181). Animals were housed in individual ventilated cages in a barrier facility proactive in environmental enrichment. *AhCre* and *p21*^*−/−*^ (*Cdkn1a*^*tm1Led*^) mice were sourced from Owen Sansom (Cancer Research UK, Beatson Institute, Glasgow)^28^,^29^, *Fh*^*fl/fl*^ mice kindly provided by Iain Tomlinson (University of Oxford)^19^ and all were maintained on a C57Bl6 background. Experimental mice (mixed sex) were aged to 12–18 months and no difference between male and female animals was observed.

### Cell culture

*Fh1*^*fl/fl*^*, Fh1*^*Δ/Δ*^, UOK262 and UOK262pFH cell lines were obtained and cultured as previously described^8^. In brief, all cell lines were cultured in DMEM supplemented with 10% fetal bovine serum and 2 mM glutamine. The mouse cell lines were also supplemented with 1 mM pyruvate and 50 μg ml^−1^ uridine.

### Immunohistochemistry

Mouse kidneys were fixed in 10% neutral buffered formalin and paraffin embedded. Sections (4 μm) were cut and stained for Ki67 (1:100 Thermo RM-9106), Cytokeratin (Cytokeratin, Pan Ab-1, Mouse Monoclonal Antibody 1:100, Thermo, MS-34) and p21 (1:500 Santa Cruz, sc471).

### Chemical reagents

Unless otherwise stated, all chemical compounds were purchased form Sigma (Gillingham, Dorset, UK). ^13^C-labelled compounds were purchased by Cambridge Isotopoes (Tewksbury, MA).

### The transcriptomics signature of FH-deficient cells

To examine whether the *Fh1*^*Δ/Δ*^ cells are under oxidative stress based on their gene expression (http://www.ncbi.nlm.nih.gov/geo/query/acc.cgi?acc=GSE63438) we identified genes whose expression is significantly higher in the *Fh1*^*Δ/Δ*^ cells compared with the *Fh1*^*fl/fl*^ cells (false discovery rate-corrected *P*-values <0.05) (ref. 8). We then assembled a set of 281 genes that are known to be involved in the response to oxidative stress. This set includes genes that are annotated with one of the 32 gene ontology terms which are child terms of the gene ontology term response to oxidative stress^30^ (for example, response to ROS, response to superoxide, response to oxygen radical). We examined if the genes that are significantly upregulated in the *Fh1*^*Δ/Δ*^ cells compared with the *Fh1*^*fl/fl*^ cells are enriched with this set of oxidative stress genes, via a hypergeometric enrichment test. We then examined if FH deficiency induces a gene expression signature of oxidative stress also in clinical samples. To this end, we analysed a previously defined set of 1,397 genes that are upregulated in HLRCC relative to normal kidney tissues^7^, and tested if this set is enriched with oxidative stress genes by performing a hypergeometric enrichment test.

### Metabolomic extraction of mouse tissue

Kidneys were excised and immediately processed for metabolomics extraction. Equal amounts of renal tissue were lysed in 250 μl of extraction solution per each 10 mg of tissue in Precellysis vials following manufacturer’s instruction. The extraction solution contained 30% acetonitrile, 50% methanol and 20% water. The suspension thus generated was immediately centrifuged at 16,000 *g* for 15 min at 0 °C. The supernatant was then submitted to LC-MS metabolomic analysis.

### Metabolomic extraction of cell lines

Cells (5 × 10^5^) were plated onto six-well plates and cultured in standard medium for 24 h. For the intracellular metabolomic analysis, cells were quickly washed for three times with PBS to remove contaminations from the media. The PBS was thoroughly aspirated and cells were lysed by adding a precooled ES. The cell number was counted in a parallel control dish, and cells were lysed in 1 ml of ES per 1 × 10^6^ cells. The cell lysates were vortexed for 5 min at 4 °C and immediately centrifuged at 16,000*g* for 15 min at 0 °C. The supernatants were collected and analysed by LC-MS. For the metabolomic extraction of spent media, 50 μl of cell media were deproteinized by 1:16 dilution in a solution composed of 80% acetonitrile and 20% water. The supernatants were then processed as described above. Fresh medium without cells was incubated in the same experimental conditions and used as a reference.

### LC-MS metabolomic analysis

For the LC separation, column A was the Sequant Zic-Hilic (150 mm × 4.6 mm, internal diameter (i.d.) 5 μm) with a guard column (20 mm × 2.1 mm i.d. 5 μm) from HiChrom, Reading, UK. Mobile phase A: 0.1% formic acid v/v in water. Mobile B: 0.1% formic acid v/v in acetonitrile. The flow rate was kept at 300 μl min^−1^ and gradient was as follows: 0 min 80% of B, 12 min 50% of B, 26 min 50% of B, 28 min 20% of B, 36 min 20% of B, 37–45 min 80% of B. Column B was the sequant Zic-pHilic (150 mm × 2.1 mm i.d. 5 μm) with the guard column (20 mm × 2.1 mm i.d. 5 μm) from HiChrom, Reading, UK. Mobile phase C: 20 mM ammonium carbonate plus 0.1% ammonia hydroxide in water. Mobile phase D: acetonitrile. The flow rate was kept at 100 μl min^−1^ and gradient as follow: 0 min 80% of D, 30 min 20% of D, 31 min 80% of D, 45 min 80% of D. The mass spectrometer (Thermo Exactive Orbitrap) was operated in a polarity switching mode.

### ^13^C-glutamine and cystine labelling experiments

Cells (5 × 10^5^) were plated onto six-well plates and cultured in standard medium for 12 h. The medium was then replaced by fresh medium supplemented with either 2 mM U-^13^C-Glutamine or 0.2 mM ^13^C_2_-Cystine for the indicated time.

### Orbitrap-Velos MS^2^ fragmentation

The S-(1, 2-Dicarboxyethyl) GSH standard (H-1556.1000, Bachem) was dissolved in water and diluted to 40 μg ml^−1^ with a 50% mixture of acetonitrile and water. An Orbitrap-Velos mass spectrometer was used in the negative mode, with HCD fragmentation MS^2^ energy of 50 unit.

### NMR measurement

Proton and two-dimensional pfgCOSY as well as pfgTOCSY were measured for standards and samples on an ECX 400 Jeol NMR equipped with a pulse-field gradient autotune 40TH5AT/FG broadband high-sensitivity probe. ^1^H NMR (400 MHz, Methanol-*d4*) *δ* 3.98 (s, 2H), 3.85–3.71 (m, 2H), 3.38 (s, 1H), 2.97 (dq, *J*=18.1, 9.3 Hz, 3H), 2.75 (dd, *J*=17.1, 5.9 Hz, 3H), 2.72–2.58 (m, 1H), 2.22 (m, 1H).

### Bioinformatics processing and statistical analysis of the metabolomic data

The metabolomics analyses obtained from cells and media extractions were log2 transformed and the replicates normalized using quantile normalization. Then, a multi-factor analysis of variance and multiple test correction were used to identify metabolites significantly different between experimental groups. Analyses were performed using Partek Genomics Suite Software, version 6.5 Copyright 2010 and R version 2.14.0.

### *In vitro* succination reaction

GSH (2 mM) and 8 mM ^13^C_2_-fumarate were mixed in 2 ml of a solution composed of 50 mM NaCl, 20 mM Tris and 0.1% Triton-X-100, pH=7.4, 4 °C for 8 h in the presence or absence of cell lysate obtained from 2 × 10^6^ *Fh1*^*Δ/Δ*^ cells previously heat inactivated (100 °C for 1 h) or left untreated. Protease inhibitor cocktail (Sigma) was used following manufacturer’s instructions. At the end of the experiment, the mixture was quenched using the extraction solution for metabolomics (see above) and the extracts were spun down at maximum speed for 10 min. The supernatant was subsequently analysed by LC-MS.

### DMF treatment

Cells (5 × 10^5^) were plated onto six-well plates and cultured in standard medium for 24 h. Then the cell culture medium was replaced with medium supplemented with the indicated concentrations of DMF for the indicated time. Cells were then harvested and extracted as described above.

### GSH GR assay

SuccinicGSH (0.2 mM, purchased from BACHEM) and GSSG (2 mM) were separately dissolved in a solution containing 50 mM Tris and 5 mM EDTA. The pH was adjusted at 7.8 to mimic the pH of the mitochondrial matrix. Substrates were added to a solution of 250 μM NADPH and 0.05 U ml^−1^ GSH GR. Metabolite production was monitored by LC-MS.

### qPCR experiments

mRNA was extracted as previously described^8^. In brief, RNA was extracted from cells of the indicated genotype using the Qiagen RNAeasy RNA extraction kit (Qiagen, Manchester, UK) following the manufacturer’s instructions.mRNA (1 μg) was retrotranscribed into cDNA using High Capacity RNA-to-cDNA kit (Life Technologies, Paisley, UK). qPCR reaction was performed using 200 ng of cDNA, the indicated primers and fast Sybr green mastermix (Life Technologies). Primers were designed using ROCHE universal probe library primer design tool (version 2.45 and 2.48) and the sequences were as follow:

p21 FW: 5′- GAGGGCTAAGGCCGAAGATG -3′, RV: 5′- GCAGACCAGCCTGACAGATTTC -3′

Rpl34 FW: 5′- GTGAGGGACGAGGAGAGGTG -3′, RV: 5′- ACCATCCTGAGTGCCGCTTC -3′

Slc7a11 FW: 5′- TGGGTGGAACTGCTCGTAAT -3′, RV: 5′- AGGATGTAGCGTCCAAATGC -3′

DDIT3 FW: 5- GCGACAGAGCCAGAATAACA -3′, RV 5′- GATGCACTTCCTTCTGGAACA -3′

PTGS1 FV: 5′- CCTCTTTCCAGGAGCTCACA -3′, RV: 5′- TCGATGTCACCGTACAGCTC -3′

PYCR1 FW: 5′- CTGACCATGTCAAGCCACTG -3′, RV 5′- TTCCCAGGGATAGCTGAGGT -3′

NQO1 FW: 5′- AGCGTTCGGTATTACGATCC -3′, RV: 5′- AGTACAATCAGGGCTCTTCTCG -3′

ABCB1A FW: 5′- GGGCATTTACTTCAAACTTGTCA -3′, RV: 5′- TTTACAAGCTTCATTTCCTAATTCAA -3′

GCLC FW: 5′- ATGATAGAACACGGGAGGAGAG -3′, RV: 5′- TGATCCTAAAGCGATTGTTCTTC -3′

HMOX1 FW: 5′- gtcaagcacagggtgacaga -3′ RV 5′- atcacctgcagctcctcaaa -3′

For the qPCR reactions, 0.5 μM primers were used—1 μl of Fast Sybr green gene expression master mix; 1 μl of each primers and a 4 μl of a 1:10 dilution of cDNA in a final volume of 20 μl were used. Real-time PCR was performed in the 7500 Fast Real-Time PCR System (Life Technologies) using the fast Sybrgreen programme and expression levels of the indicated genes were calculated using the ΔΔCt method by the appropriate function of the software using actin as calibrator.

### Primary kidney cells isolation and infection

Primary kidney epithelial cells were isolated from *Fh1*^*+/+*^ and *Fh1*^*fl/fl*^ p5 mice as described previously^31^ and cultured in DMEM supplemented with 10% fetal bovine serum, 2 mM glutamine and 1 mmol l^−1^ pyruvate. Cells were infected with Cre recombinase-encoding adenovirus (Ad5-CMV-Cre-GFP, Vector Development Laboratory) using 200 p.f.u. per cell. After 2 days, cells were transferred on 24-well plates for growth curve determination and SA-β-Gal Staining.

### Lentiviral infection of IMR90

The viral supernatant for infection was obtained from the filtered growth media of the packaging cells HEK293T transfected with 3 μg psPAX, 1 μg pVSVG, 4 μg of short hairpin RNA (shRNA) constructs and 24 μl of Lipofectamine 2000 (Life Technology). Cells (3 × 10^5^) were then plated on six-well plates and infected with the viral supernatant in the presence of 4 μg ml^−1^ polybrene. After two days, the medium was replaced with selection medium containing 1 μg·ml^−1^ puromycin. The expression of the shRNA constructs was induced by incubating cells with 2 μg ml^−1^ doxycyclin. The shRNA sequences were purchased from Thermoscientific (NTC: # RHS4743; sh #2 V3THS_324847 and sh#3: V3THS_324846)

### Senescence-associated (SA)-β-Gal staining

For mouse epithelial kidney cells, 4 × 10^4^ cells were treated for 2 days with 50 μM DMF and a Senescence β-Galactosidase Staining Kit (Cell Signaling Technology) was used. The staining was performed according to manufacturer’s instructions and images were acquired with a Zeiss Axiovert 200 M microscope. To quantify SA-β-gal positive cells, cells were counted in four random fields in each of the triplicates wells.

For IMR90, 4 × 10^4^ cells were plated onto coverslips in a 24-well plate. The day after the medium was replaced with medium containing either DMF or *N*-acetyl cysteine or a combination of the two drugs. The medium was replenished every day for a total of 5 days of treatment. At the end of the experiment, cells were washed in PBS and fixed with 0.5% glutaraldehyde solution (in PBS) for 15 min at room temperature. Immediately after cells were washed twice with 1 mM MgCl_2_ in PBS (pH 6.0) and incubated with a staining solution composed of 1 mg ml^−1^ 5-Bromo-4-chloro-3-indolyl-β-D-galactopyranoside (prepared in *N*,*N*-dimethylformamide), 0.12 mM K_3_Fe(CN)_6_, 0.12 mM K_4_Fe(CN)_6_ and 1 mM MgCl_2_/PBS (pH 6.0) at 37 °C overnight in CO_2_-free incubator. The day after, cells were washed three times with deionized water and imaged with a Zeiss Axiovert 40 CFL microscope using a × 10 objective.

### Cell proliferation

IMR90 (4 × 10^4^) were plated in a 24-well plate and placed onto the stage of the Incucyte Live Content Imaging instrument. Nine fields for each well were collected every 2 hours for the first day and then every hour for the following days. Cell proliferation was automatically determined from bright-field images by calculating cell confluence at different time points, using the appropriate function of the Incucyte software. Cell media was replaced daily with media supplemented with the indicated drugs.

### Generation of the metabolic models of *Fh1* ^*Δ/Δ*^ and *Fh1* ^*fl/fl*^ cells

To investigate the metabolic changes that occur due to FH-inactivation metabolic models of Fh1^*Δ/Δ*^ and Fh1^*fl/fl*^, cell lines were constructed based on their transcriptomics and uptake-secretion metabolomics. The construction was conducted in two stages. In the first stage, each model was constrained to obtain an optimal fit to the gene expression signature of the cell line by solving a Mixed Integer Linear Programming problem, via the integrative Metabolic Analysis Tool (iMAT)^32^,^33^. In the second stage, each model was further constrained to obtain an optimal fit to the measured uptake-secretion flux rates. The latter was achieved by forming the following problem:




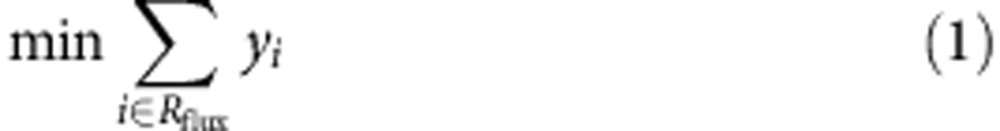




subject to




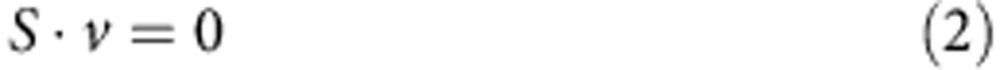







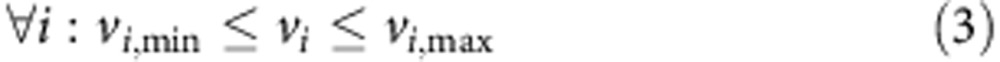







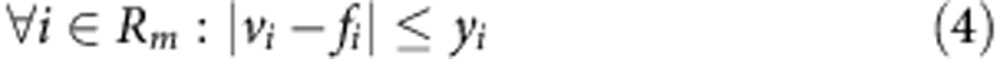




Where **v** is the flux vector and *S* is an *m* × *n* stoichiometric matrix, in which *m* is the number of metabolites and *n* is the number of reactions. The mass balance constraint is enforced by equation (2). Thermodynamic and enzyme capacity constraints are imposed in equation (3), by setting *v*_*i*,min_ and *v*_*i*,max_ as a lower and upper bound of the flux values through reaction *i*, respectively. In equation (4), *R*_*m*_ includes the set of measured reactions; for each measured reaction *i*, the variables *y*_*i*_ represents the deviation of its computed flux rate, denoted as *v*_*i*_, from its measured flux rate, denoted as *f*_*i*_. The objective function equation (1) is to minimize the deviation of the computational flux rates from the measured flux rates. To avoid the non-linear constraint imposed in equation (4) two variables, *y*^*+*^ and *y*^*−*^, are introduced to convert the problem to its equivalent linear form.




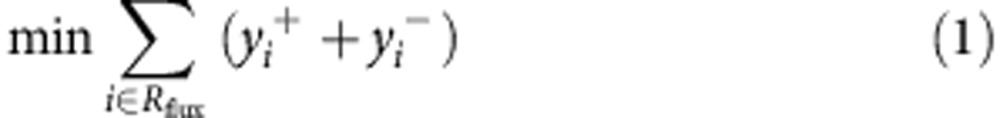




subject to




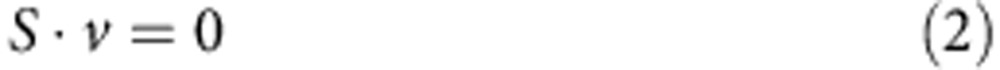







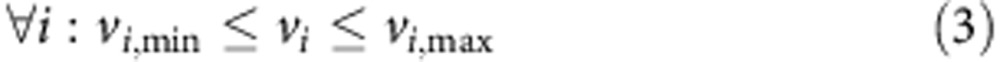







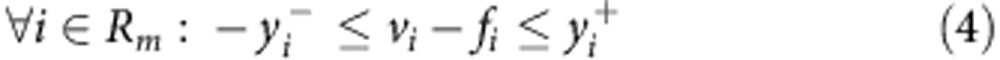







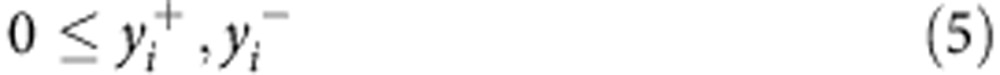




A solution found by the Mixed Integer Linear Programming solver to the iMAT problem and then by a Linear Programming solver to the problem formulated above is guaranteed to be optimal, meaning, to maximize the similarity to the expression and then, under the optimal fit to the transcriptomics to obtain a maximal fit to the flux measurements. However, the solution is not necessarily unique, as a space of alternative optimal solutions may exist. The space of optimal solutions represents various steady-state flux distributions attaining the same similarity with the expression data and flux measurements. To account for these alternative solutions, the optimal similarity to the expression and flux measurements is calculated. The model is then constrained to attain this optimal similarity, which reduces the solution space significantly.

To explore the optimal solution space of the model, that is, the feasible metabolic states which maximize the fit to the experimental data, flux variability analysis (FVA)^34^ was employed. FVA obtains for each reaction its minimal and maximal attainable flux, subject to the aforementioned constraints. In addition, by applying FVA, the maximal production rate of every metabolite in the models was computed; in this case, the production of a metabolite was defined as the sum of its production rates in all the different compartments of the model (for example, mitochondria, cytoplasm). For each metabolic pathway, the median fold-change in the production of its metabolites was computed based on the pathway definition of ref. 13. Fianlly, the redox potential of the models was estimated by computing their capacity to produce NADH and NADPH from NAD^+^ NADP^+^, respectively.

The metabolic model of the FH-deficient cells were utilized to examine the dependency of GSH production on the uptake-secretion rates of different metabolites, including amino acids and main carbon sources. First, for each metabolite in this set, the feasible flux range of its exchange reaction was computed via FVA. An exchange reaction represents the secretion or uptake of a given metabolite. A positive flux rate through an exchange reaction denotes secretion and a negative flux rate denotes uptake. Next, for every one of the metabolites the model was constrained to carry out an increasing flux of its exchange reaction, which is within its feasible flux range. Under each of these constraints, the maximal production of reduced GSH in the model was computed. Finally, for each metabolite, the Spearman correlation between the upper bounds of the metabolite exchange reaction and the pertaining maximal production rates of GSH was computed. A positive correlation, as found between GSH production and arginine, denotes that a higher flux rate through the exchange reaction of the metabolite—less uptake or more secretion—enables the model to produce GSH more rapidly. Likewise, a negative correlation, as found between GSH production and cystine, denotes that a lower flux rate through the exchange reaction of the metabolite—more uptake or less secretion—enables the model to produce GSH more rapidly.

The ability of the models to predict the measured flux rates was examined in a leave-one-out cross-validation process. Every iteration, the models were regenerated based on their gene expression and a partial set of the metabolic uptake and secretion rates. These models were then used to compute via FVA the maximal and minimal flux rate of the exchange reaction whose flux measurement was omitted from the construction process. Significant correlation was found between the measured and predicted flux rates (Supplementary Table 2).

Following the validation of the models, they were utilized to systematically explore the metabolic differences between the Fh1^*Δ/Δ*^ and Fh1^*fl/fl*^ cells. The capacity of the two models to produce each of their 1,491 metabolites was computed. The FH-deficient model was found to have a higher capacity to produce 43 metabolites (Supplementary Fig. 1b). Among the top ones are four derivatives of GSH: Oxidized GSH, reduced GSH, (R)-S-Lactoylgutathione and S-Formylglutathione (Supplementary Fig. 1b). These results suggest that FH deficiency induces oxidative stress. To further examine this hypothesis, the ability of the Fh1^*fl/fl*^ and Fh1^*Δ/Δ*^ models to produce reducing power in the form of NADH and NADPH was computed. Indeed, the Fh1^*fl/fl*^ model was found to have a significantly higher capacity to produce NADH and NADPH, compared with the Fh1^*Δ/Δ*^ model.

Following the discovery of GSH succination in the FH-deficient cells, the models were reconstructed with GSH succination (see next section). According to the updated models, GSH succination further severely limits NADPH and NADH production in Fh1^*Δ/Δ*^ cells. This strong coupling between GSH succination and decreased NADH/NADPH production is highly notable, since the activity of almost all other reactions in the Fh1^*Δ/Δ*^ model does not entail such a decrease (Supplementary Fig. 1c, Wilcoxon ranksum *P* value of 7.91e−04). Further computational analysis revealed that this coupling arises since, in the Fh1^*Δ/Δ*^ cells, GSH synthesis depends on cystine uptake, rather than on methionine uptake (Supplementary Fig. 3b). The synthesis of GSH from cystine causes the oxidation of two GSH molecules. As reducing them requires NADPH, it imposes a constraint to activate pathways that contribute to NADPH production.

GSH succination was introduced to the models by adding the following reactions

1. Mitochondrial reaction: reduced GSH [m]+fumarate [m]→succinicGSH
2. Cytoplasmatic reaction: reduced GSH [c]+fumarate [c]→succinicGSH
3. Sink reaction: succinicGSH

Reaction (3) denotes the secretion of succinicGSH. In the FH-deficient model, this reaction was constrained to carry a non-zero flux. Note that some of the succinicGSH may be metabolized via additional metabolic reactions which are yet to be discovered, as opposed to secreted. However, currently, the models cannot account for such reactions.

## Additional information

**How to cite this article:** Zheng, L. *et al*. Fumarate induces redox-dependent senescence by modifying glutathione metabolism. *Nat. Commun.* 6:6001 doi: 10.1038/ncomms7001 (2015).

## Supplementary Material

Supplementary Information Supplementary Figures 1-6, Supplementary Tables 1-3 and Supplementary References
