# Supplementary material for: Fumarate induces redox-dependent senescence by modifying glutathione metabolism
Source: Nat Commun. 2015 Jan 23;6:6001. doi: 10.1038/ncomms7001 (PMC4340546; doi:10.1038/ncomms7001)
Supplement: Supplementary Information — Supplementary Figures 1-6, Supplementary Tables 1-3 and Supplementary References [file ncomms7001-s1.pdf]

Supplementary Figure 1

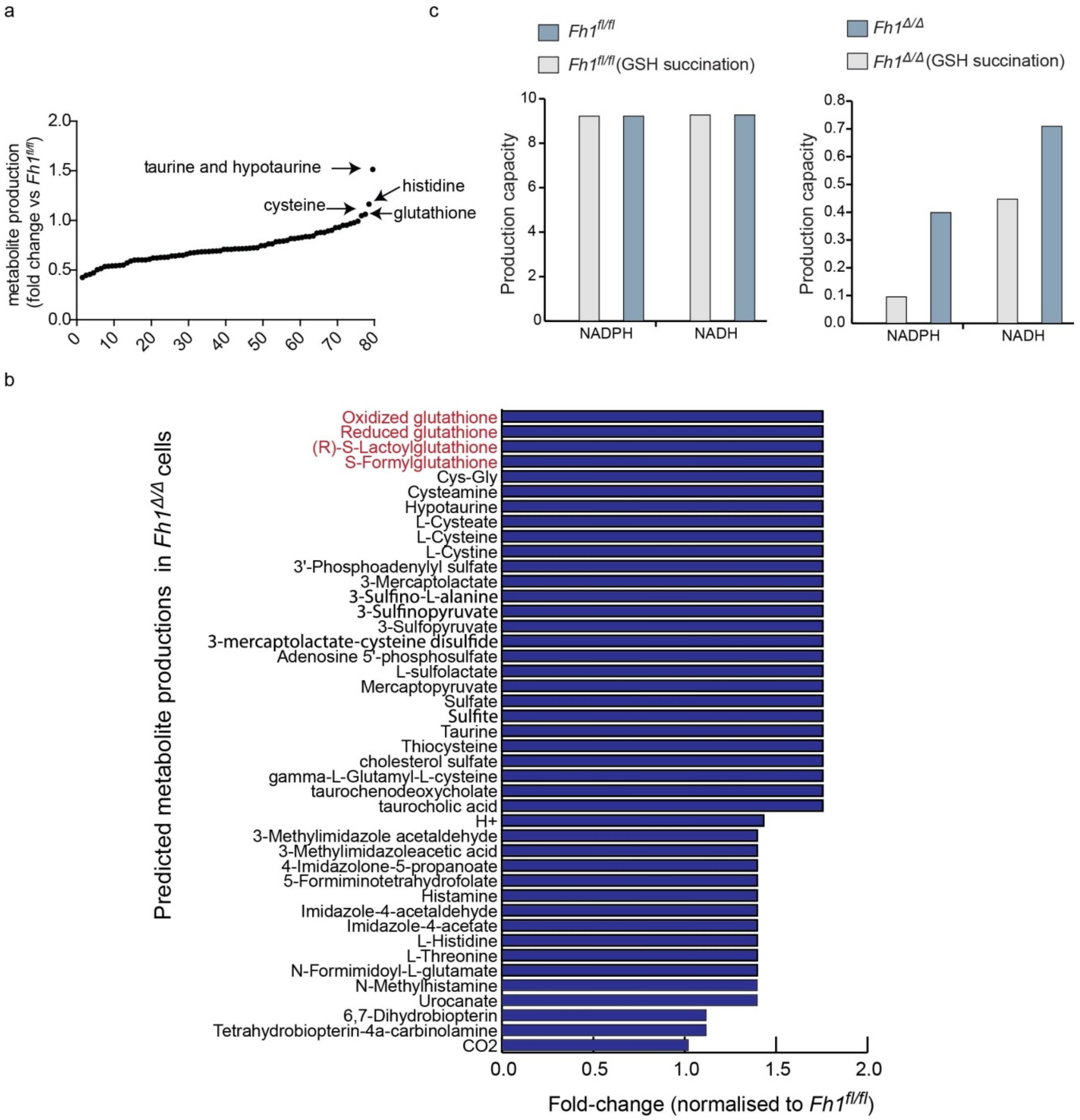

Supplementary Figure 1: Antioxidant signature in FH-deficient cells. (a) The predicted fold-change of the rates of metabolic pathways related to the indicated metabolite production in the FH-deficient computational model compared to the wt model. (b) The predicted fold-change in the capacity of FH-deficient cells to produce different metabolites as compared to control. Only the metabolites that their predicted production is induced are shown. (c) Predicted NADPH and NADH production capacity in the $Fh1^{fl/fl}$ and $Fh1^{\Delta/\Delta}$ models, with and without a constraint to include GSH succination.

Supplementary Figure 2

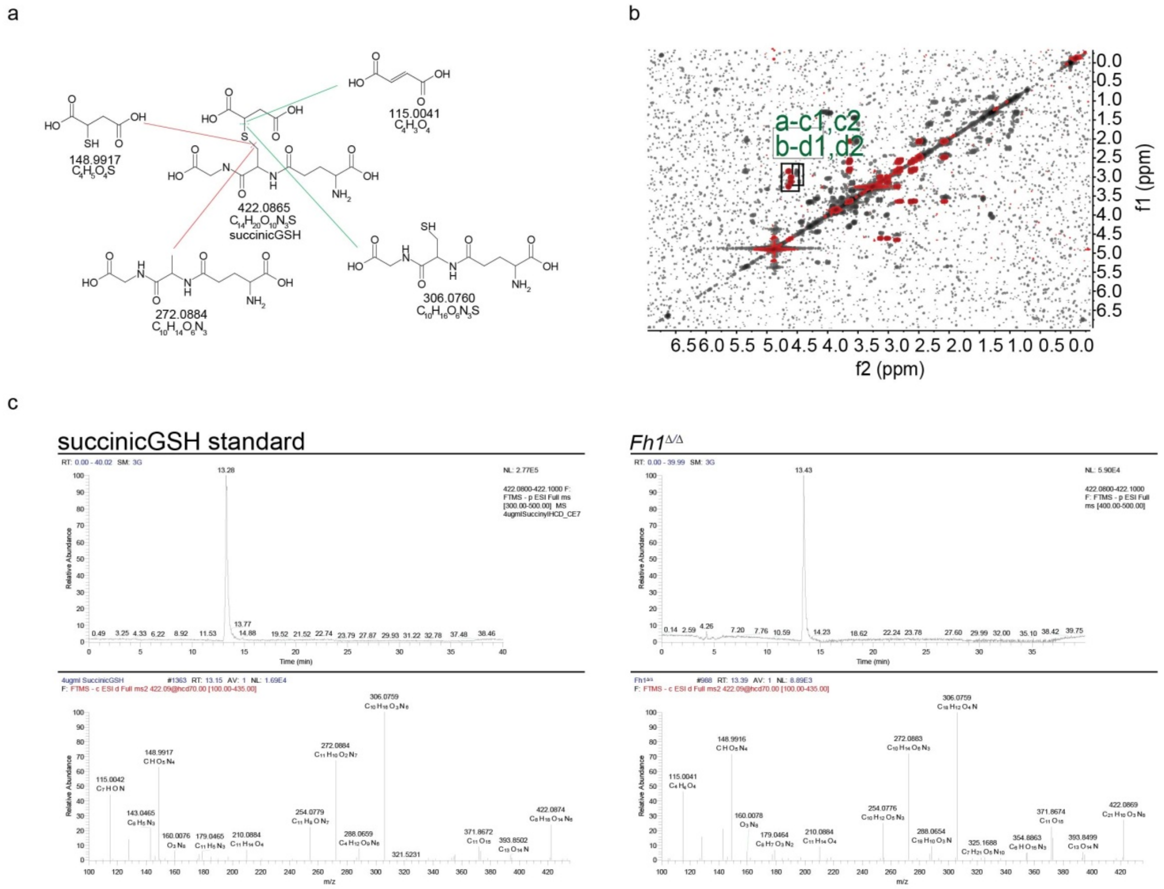

Supplementary Figure 2: Structural elucidation of succinicGSH.

(a) Chemical structure of succinicGSH and its daughter ions identified by LC-MS/MS. (b) NMR pulse field gradient correlation spectroscopy (pfgCOSY) and pulse field gradient total correlation spectroscopy (pfgTOCSY) of chemically-produced succinicGSH (red) superimposed on spectra obtained from FH-deficient cell extracts (black). (c) LC-MS/MS spectra of succinicGSH standard (left) and the identified peak in Fh1-deficient cells (right).

Supplementary Figure 3

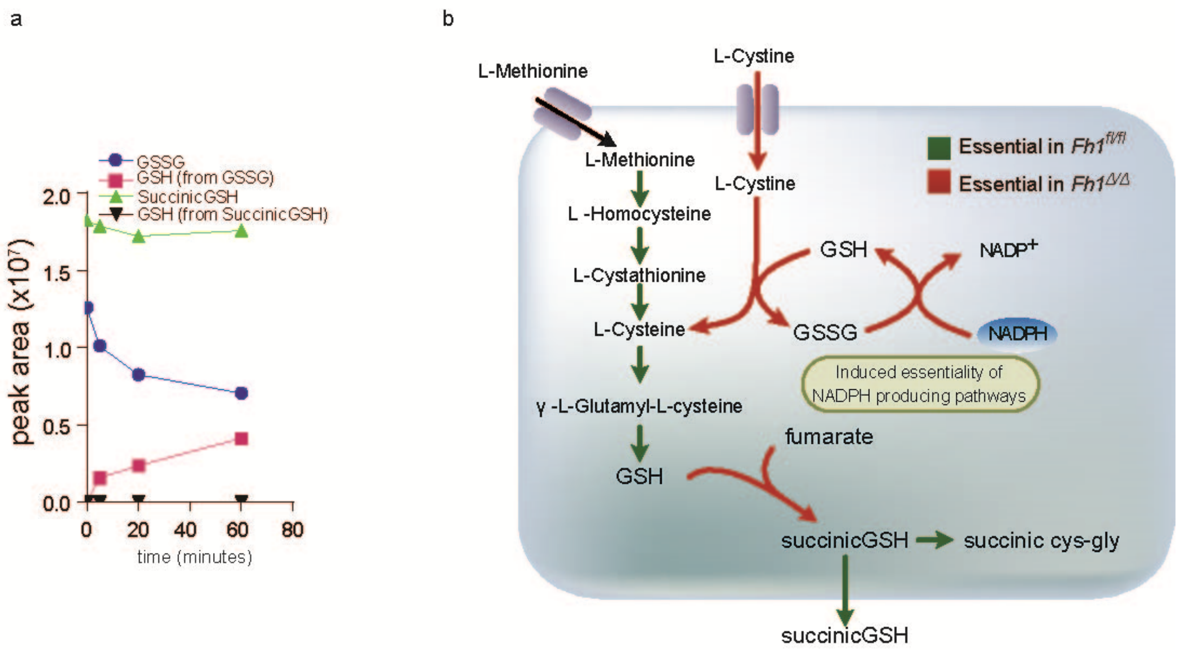

Supplementary Figure 3: The succination of GSH reshapes the generation of NADPH and induces oxidative stress.. (a) The relative levels of GSH produced *in vitro* by glutathione reductase in the presence of NADPH from the indicated substrates, GSSG or SuccinicGSH were assessed by LC-MS at the indicated time points. (b) A model of GSH synthesis and succination in FH-deficient cells. GSH synthesis is induced, and relies mainly on cystine uptake and reduction to cysteine. In this model GSH succination reshapes the production of reducing power (NADPH).

Supplementary Figure 4

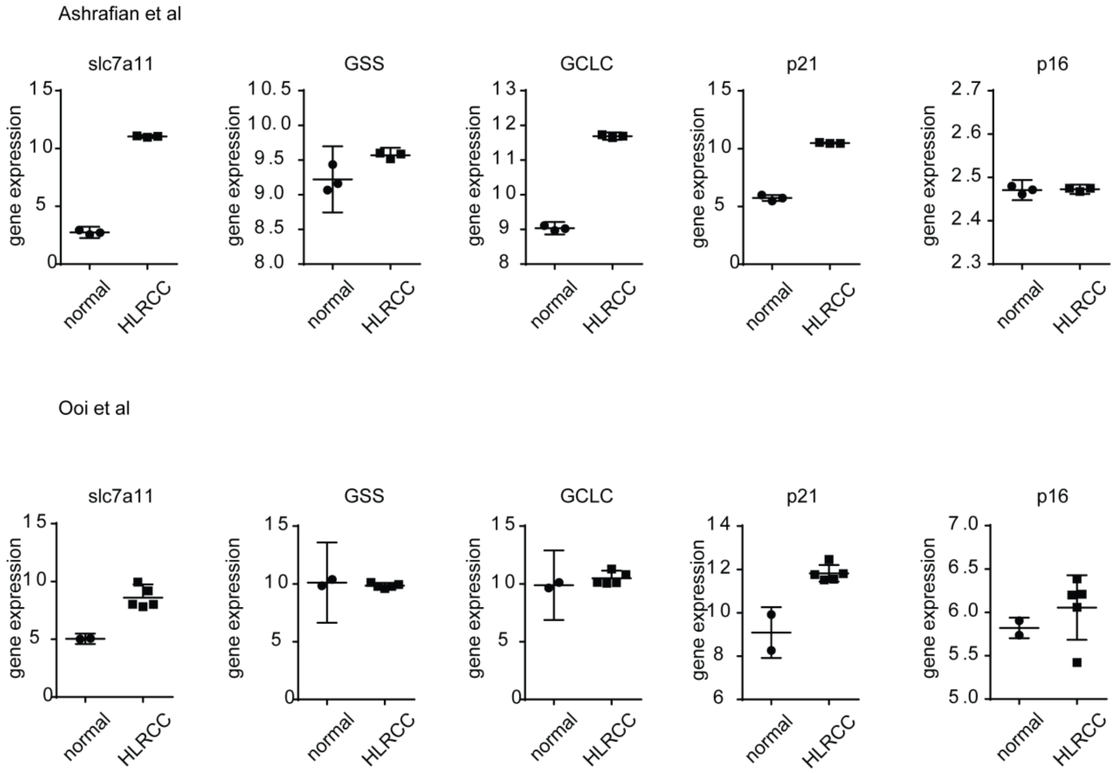

Supplementary Figure 4: Glutathione biosynthesis and senescence transcriptional signatures are upregulated in HLRCC patients. Gene expressions of Slc7a11 (cystine transporter) GSS, GCL (GSH biosynthesis enzymes), p21 and p16 are extracted from the transcriptomics profile of HLRCC patients in the indicated studies, Ashrafian[1] et al and Ooi et al[2]. The error bars represent 95% Confidence Interval.

Supplementary Figure 5

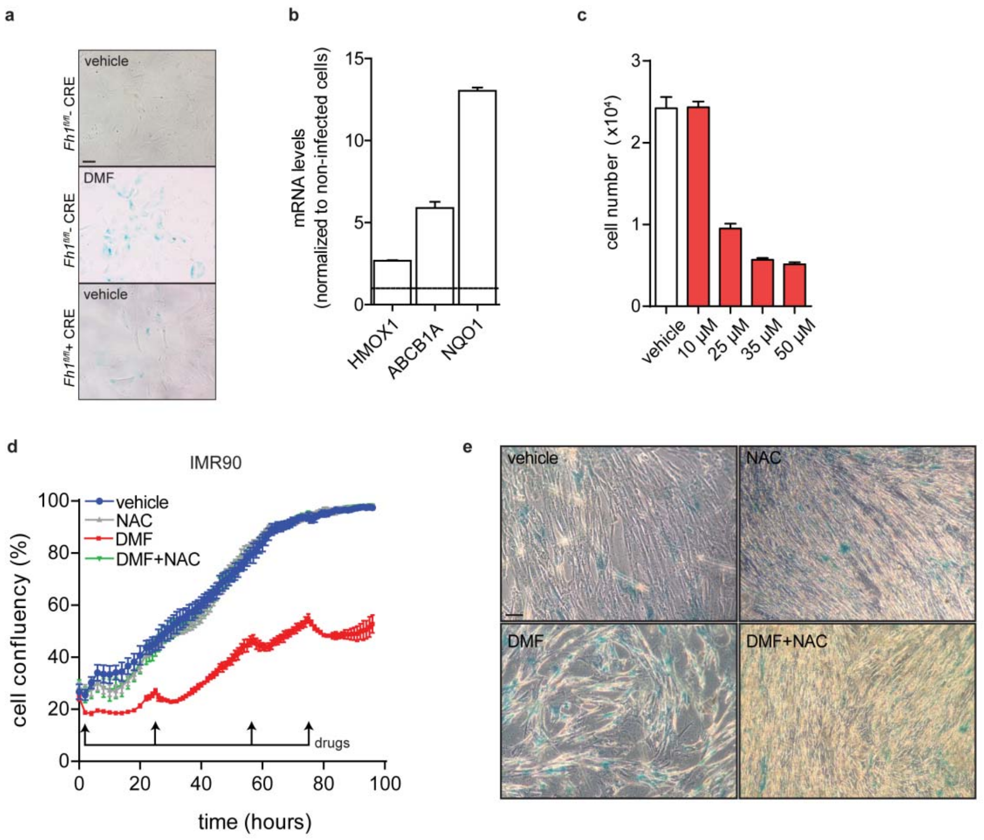

Supplementary Figure 5: GSH succination leads to redox stress-induced senescence in primary kidney cells and human diploid fibroblasts

(a) Representative pictures of senescence-associated β-galactosidase activity induced by FH ablation or DMF in primary kidney cells. (b) The ablation of FH in kidney cells results in the activation of the antioxidant response genes HMOX1, ABCB1A and NQO1 (n=3). (c-d) DMF induces proliferation arrest of primary epithelial kidney cells (c) and of IMR90 cells (d), which can be recovered by NAC. (e) Representative pictures of senescence-associated β-galactosidase activity induced by DMF in IMR90. Results were obtained from 3 independent cultures and expressed as average ± s.e.m. Scale bar represents 100 µm.

Supplementary Figure 6

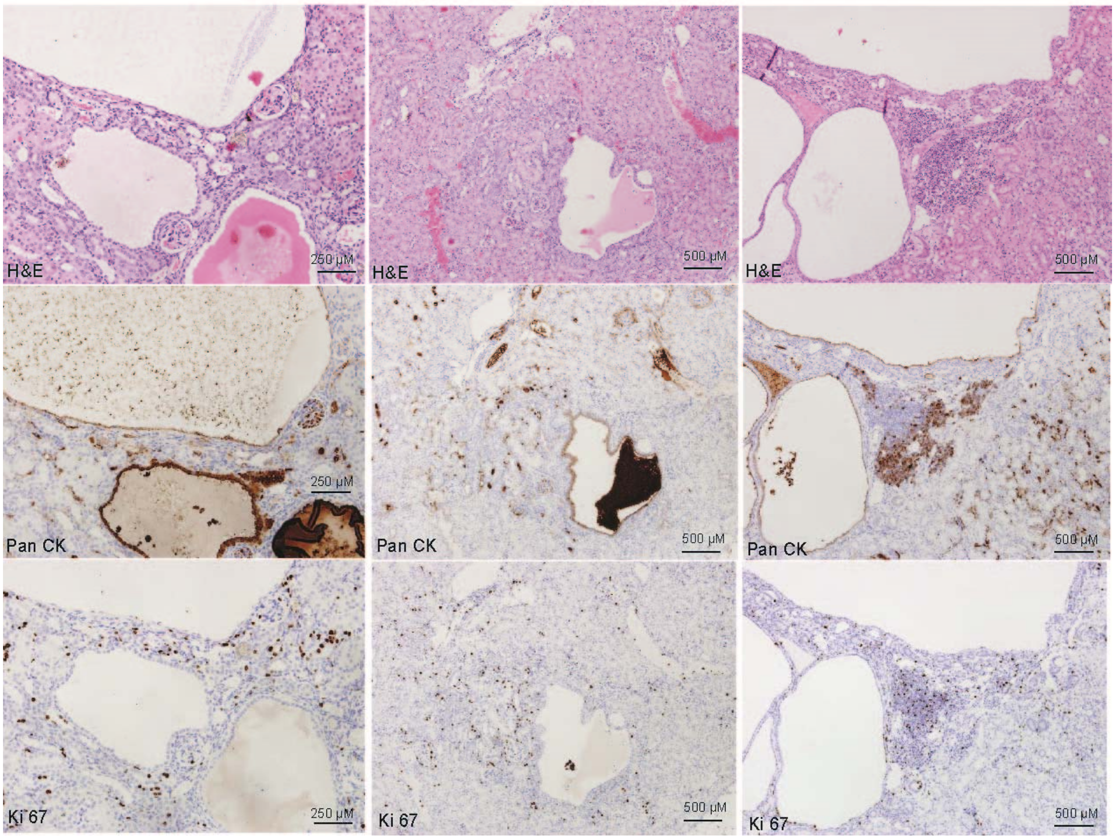

Supplementary Figure 6: Ablation of Fh1 and p21 resulted in sever hyperplasia of kidney epithelial cells. A detailed immunohistochemistry analyses of hyperplasic kidney sections of p21$^{-/-}$*Fh1*$^{fl/fl}$*AhCre*$^{+}$ mice. Sections were analysed by haematoxylin & eosin (H&E) staining, pan-cytokeratin (Pan CK) antibody for detection of epithelial origin and anti-Ki67 antibody for detection of actively proliferating cells. Scale bar on the left represents 250 µm; on the middle and right represents 500 µm

Supplementary Table 1 FH Quantitative medium exchanging rate.

	Fh1$^{+/+}$		*Fh1*$^{\Delta/\Delta}$	
Metabolites*	*Mean*	*Std dev*	*Mean*	*Std dev*
Fum	0.604	0.042	281.261	10.850
Mal	2.674	0.334	1.451	0.406
Pyr	-148.225	0.576	-389.099	0.152
Lac	2513.801	35.208	6760.887	73.873
Glucose	-1738.855	184.241	-3990.470	288.300
Hexose-Phosphate	0.442	0.074	0.943	0.086
a-KG	6.403	0.364	1.470	0.231
Suc	2.595	0.378	28.827	1.449
Phe	-4.454	2.123	9.927	3.037
Ala	72.909	4.036	133.797	4.810
Gly	52.161	8.995	113.549	3.927
Argininosuccinate	-0.015	0.000	12.617	0.447
Arg	-6.594	2.341	1.800	0.498
Asp	2.837	0.493	5.583	0.178
Asn	-2.046	0.039	7.192	0.587
Gln	-272.674	1.957	-359.541	10.174
Glu	42.493	2.548	65.659	2.230
Tyr	-5.547	0.456	4.063	1.459
His	1.555	1.845	11.861	1.707
Ser	-51.279	1.028	-79.328	2.489
Met	-10.295	0.709	-1.455	1.267
Pro	24.387	2.044	11.370	1.401
Thr	-3.025	5.080	20.854	5.200
Leu/Ile	-84.592	4.269	-8.281	10.075
Trp	-1.754	0.306	1.571	0.502
Citrulline	0.167	0.072	0.290	0.265
Lys	-9.530	1.978	15.147	0.822
Ornithine	0.917	0.183	1.623	0.343
Cystine	-1.903	0.594	-10.121	1.728
*Δpmol metabolites/ µg protein/ Δt (hr)				

Results were obtained from 3 independent experiments and expressed as average ± s.d

Supplementary Table 2. The correlation between the computed and measured flux-rates.

Correlation type	Flux type	Correlation coefficient			p-value		
		WT	KO (with GSH-succination)	KO (without GSH succination)	WT	KO (with GSH-succination)	KO (without GSH succination)
Spearman	Maximal	0.456	0.569	0.485	1.26E-02	1.85E-03	8.21E-03
	Minimal	0.764	0.484	0.484	7.05E-06	8.26E-03	8.26E-03
	Average	0.617	0.570	0.551	6.59E-04	1.81E-03	2.63E-03
Pearson	Maximal	0.537	0.965	0.961	3.39E-03	1.39E-14	4.71E-14
	Minimal	0.603	0.548	0.548	9.05E-04	2.79E-03	2.79E-03
	Average	0.726	0.760	0.758	2.93E-05	8.36E-06	9.03E-06

Supplementary Table 3 The correlation between metabolite uptake-secretion rates and GSH production

Metabolite	Spearman correlation	
	Coefficient*	p-value
L-Cystine	-1	5.98E-06
L-Serine	-0.307	0.188
D-Glucose	-0.305	0.190
L-Alanine	-0.295	0.207
L-Glutamine	-0.284	0.224
Pyruvate	-0.278	0.234
L-Lactate	-0.272	0.245
Argininosuccinate	-0.271	0.247
Succinate	-0.266	0.256
2-Oxoglutarate	-0.254	0.278
L-Glutamate	-0.229	0.331
L-Proline	-0.221	0.347
Glycine	-0.220	0.350
L-Asparagine	-0.191	0.418
L-Tyrosine	0.032	0.895
L-Phenylalanine	0.633	3.39E-03
L-Methionine	0.814	1.17E-05
L-Aspartate	0.998	6.03E-06
L-Histidine	0.998	6.03E-06
L-Threonine	0.998	6.03E-06
L-Tryptophan	0.998	6.03E-06
L-Lysine	0.998	6.03E-06
Ornithine	0.998	6.03E-06
L-Arginine	1	5.98E-06

*A negative correlation denotes GSH production increases with the increase in the metabolite uptake (leading to a faster decrease in the extracellular metabolite abundance).

References

1. Ashrafian, H., et al. Expression profiling in progressive stages of fumarate-hydratase deficiency: the contribution of metabolic changes to tumorigenesis. Cancer research 70, 9153-9165 (2010).

2. Ooi, A., et al. An antioxidant response phenotype shared between hereditary and sporadic type 2 papillary renal cell carcinoma. Cancer Cell 20, 511-523 (2011).
